# PRIVATE PARTS: AGING, AI, AND THE ETHICS OF CONSENT IN SUBSCRIPTION-BASED ECONOMIES

**DOI:** 10.1093/geroni/igz038.082

**Published:** 2019-11-08

**Authors:** Kim Sawchuk

**Affiliations:** Concordia University, Montreal, Quebec, Canada

## Abstract

This paper explores Artificial Intelligence (AI) as a technological design offered to assist elder-care based on tracking individual behavior amassed in data bases that are given predictive value through algorithm-identified normative patterns. Examples are drawn from ethnographic research conducted at the 2019 Consumer Electronics Show, including document analysis of product promotions and interviews conducted with company representatives, supplemented by interviews with older adults. The paper focuses on the ethical dilemmas of privacy, security, consent, and identity in home surveillance systems and financialization of personal data in AI subscription-based services. Theoretically the argument, informed by recent discussions on the datafication of self and quantified aging, emphasizes that such a subscription-based economy exploits older individuals by sharing their lifestyle profiles, health information, economic status, and consumer preferences within powerful corporate networks such as Google and Amazon. Conclusions question how the promise of AI is being tied to the future of population aging.

